# Flood-related WASH disruption, point-of-use drinking water contamination and diarrhoea among under-five children in flood-affected communities in Ebonyi State, Nigeria

**DOI:** 10.1186/s12889-026-29000-w

**Published:** 2026-08-15

**Authors:** Onwe Paul Chikaodili

**Affiliations:** Department of Public Health, Abakaliki Study Centre, National Open University of Nigeria, Abakaliki, Ebonyi State Nigeria

**Keywords:** Diarrhoea, Drinking water contamination, *Escherichia coli*, Flood, Nigeria, WASH

## Abstract

**Background:**

Flooding disrupts water, sanitation and hygiene (WASH) systems, increasing the risk of waterborne diseases among young children. This study examined the relationships between flood-related WASH disruption, drinking water contamination and diarrhoea among under-five children in flood-displaced communities in Ebonyi State, Nigeria.

**Methods:**

A cross-sectional study was conducted among 600 households across six flood-displaced communities. Point-of-use drinking water samples were tested for *Escherichia coli* and total coliform. Chi-square tests and multivariable logistic regression were used to examine associations.

**Results:**

Most households (69.5%) reported direct flood exposure and water access was disrupted in 59.7%. *Escherichia coli* contamination was detected in 48.5% of samples and total coliforms in 53.7%. The two-week prevalence of diarrhoea among under-five children was 23.3%. Contamination was uniformly high across communities (all *p* > 0.05). Water treatment method was significantly associated with *Escherichia coli* contamination (*p* = 0.023). Multivariable analysis showed that a storage container observed to be covered during the household visit was the only significant predictor of diarrhoea (AOR = 2.01, 95% CI: 1.33–3.02, *p* = 0.001).

**Conclusion:**

Microbial contamination was widespread across the study communities. Post-collection water handling and storage appeared to be more strongly associated with diarrhoea than water source. Integrated emergency WASH responses prioritising household water treatment, safe storage and sanitation are urgently needed in flood-affected communities.

**Supplementary Information:**

The online version contains supplementary material available at https://doi.org/10.1186/s12889-026-29000-w.

## Introduction

Safe and reliable water, sanitation and hygiene (WASH) services are essential for health, human dignity and the prevention of infectious diseases [1–3]. When these services are inadequate or unavailable, the risk of enteric and other infections increases, especially among young children and other vulnerable groups [4–6]. This risk becomes greater during flooding periods, when drinking water sources may be contaminated, sanitation facilities may be damaged and routine hygiene practices may be disrupted [7, 8].

Flood-related displacement creates further difficulties for affected households [9]. Families are forced to move into temporary shelters or overcrowded settlements, such as schools, open fields, churches and informal settlements, where access to safe water, toilets and handwashing facilities are often limited [2, 7, 9]. Under these conditions, diarrhoeal diseases can spread quickly, particularly among children under-five years of age [4]. These children are more likely to suffer dehydration, malnutrition and severe illness during flood periods [6]. For this reason, understanding WASH conditions in flood-displaced settings is important for both emergency response and longer-term public health planning [10, 11]. Although global access to WASH services has improved, major gaps remain [1, 12, 13]. Large populations still lack safe drinking water, adequate sanitation and basic hygiene services [1, 2, 10, 14, 15]. These deficits are most severe in low- and middle-income countries, where weak infrastructure, poor sanitation and faecal contamination of water continue to drive a high burden of diarrhoeal disease [5, 16, 17]. Climate-related disasters, including flooding, are worsening these conditions by increasing water insecurity and further weakening already fragile WASH systems [14, 18].

Nigeria frequently experiences flooding that affects livelihoods, infrastructure and public health across many states [7, 19, 20]. These events often disrupt water supply systems, damage sanitation facilities and increase exposure to contaminated water, thereby raising the risk of diarrhoeal disease and other water-related infections [7, 8, 11]. The effects are particularly important in rural and flood-prone settings such as Ebonyi State, where many households depend on wells, boreholes, streams and other decentralised water sources for their daily needs [21]. These sources are often poorly protected and may become contaminated during and after flood events through runoff, overflow of latrines, open defecation and the intrusion of polluted surface water [22]. In many communities, limited water treatment, unsafe storage practices and weak sanitation infrastructure further increase the risk of exposure to unsafe drinking water [23, 24]. As a result, households, especially those with young children, face increased vulnerability to waterborne infections and other related health consequences [19, 21, 22, 25].

Studies from Nigeria and other low-resource settings have shown that poor WASH conditions are associated with childhood diarrhoea [20–22, 24–26]. However, evidence from flood-displaced communities in Ebonyi State remains limited. In particular, few studies have jointly examined household WASH disruption, point-of-use microbiological contamination of drinking water and diarrhoea outcomes among under-five children in this setting. This is an important gap because household reports alone may not reflect the actual microbiological safety of drinking water in flood-affected communities. This study therefore aimed to examine the relationships between flood-related WASH disruption, point-of-use drinking water contamination and diarrhoea among under-five children in flood-displaced communities in Ebonyi State, Nigeria.

## Materials and methods

### Study area

**Fig. 1:**
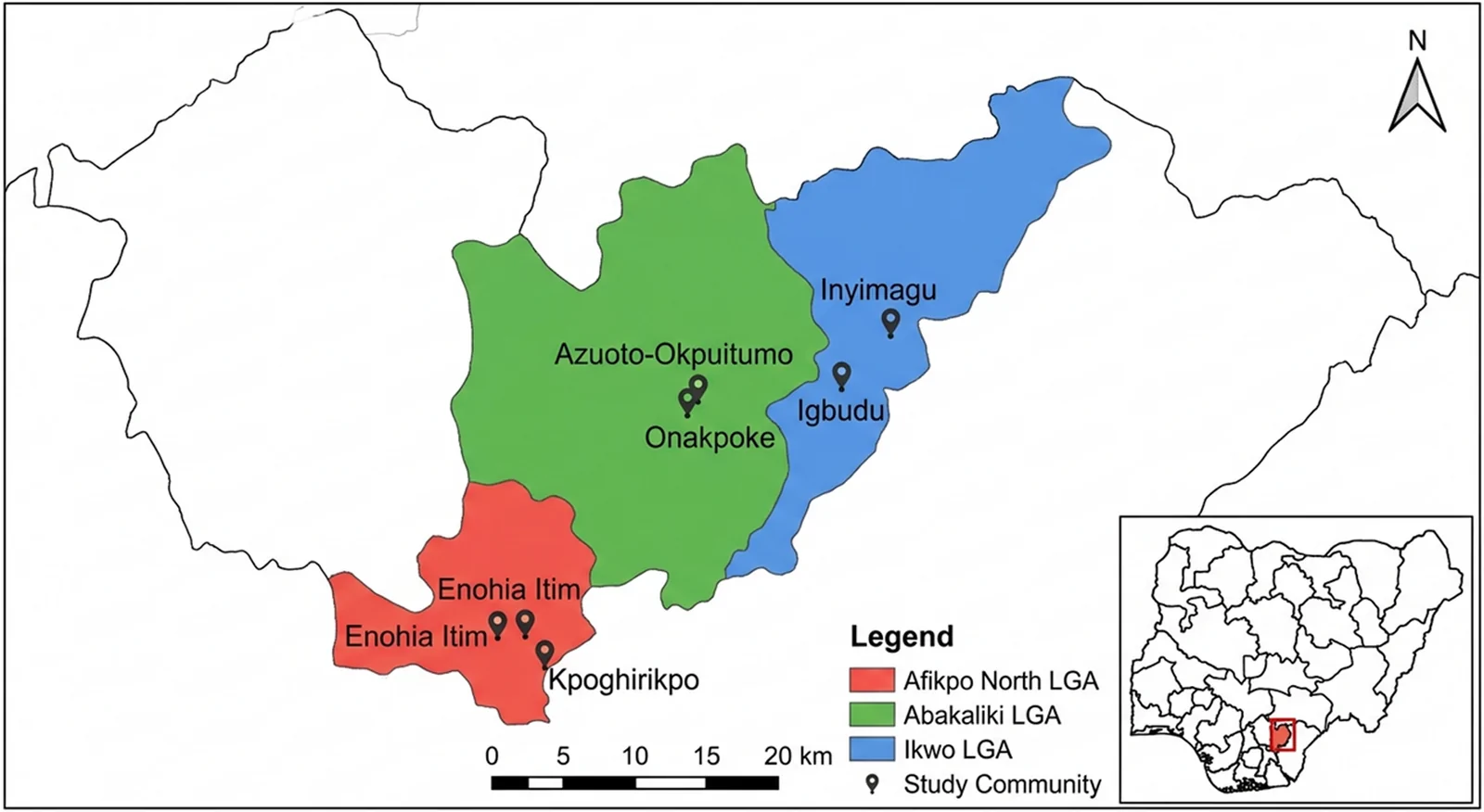
Map of the study area showing the six flood-displaced communities across three Local Government Area axes in Ebonyi State, Nigeria. Communities are grouped by LGA axis: Enohia Itim and Kpoghirikpo (Afikpo North LGA); Azuoto-Okpuitumo and Onakpoke (Abakaliki LGA); and Inyimagu and Igbudu (Ikwo LGA). The inset shows the location of Ebonyi State within Nigeria

This study was conducted in Ebonyi State, located in southeastern Nigeria. Ebonyi was created in 1996 and is divided into three senatorial zones—North, Central and South—and thirteen Local Government Areas (LGAs). The state is largely rural and agriculture is the main livelihood in many communities. Crops such as rice and cassava are widely cultivated, especially in areas influenced by the Ebonyi River and other local water bodies. Ebonyi has a tropical climate with distinct rainy and dry seasons. During the rainy season, heavy rainfall often leads to flooding, especially in low-lying and riverine communities. Flooding is a recurring environmental and public health problem in Ebonyi State, damaging houses, roads, health facilities, water sources and sanitation infrastructure [19] and disrupting access to safe water, sanitation and hygiene services [25]. In some cases, households are displaced to temporary shelters such as schools and open fields where WASH services are poor [21], increasing exposure to unsafe water and water-related diseases among young children [27]. The study was conducted in six flood-affected communities: Enohia Itim, Kpoghirikpo, Azuoto-Okpuitumo, Onakpoke, Inyimagu and Igbudu (Fig. 1), representing areas with repeated flood-related WASH disruption and environmental vulnerability.

### Study design

This study employed a community-based cross-sectional household design with microbiological assessment of point-of-use drinking water. The study was conducted in six flood-affected communities in Ebonyi State, Nigeria. Data collection was conducted during the post-flood period and took place within the same period across all study communities to ensure comparability and reduce the influence of temporal changes in water quality and WASH conditions. The quantitative component involved a household survey and laboratory testing of point-of-use drinking water samples. A total of 600 households were selected using a multistage cluster sampling approach. Structured caregiver-reported questionnaires collected information on household characteristics, flood-related WASH disruption experienced during the most recent flood event, drinking water sources, sanitation conditions, hygiene practices and recent diarrhoea among under-five children. One point-of-use drinking water sample was collected from each household for microbiological analysis, producing 600 water samples, with 100 from each community to allow comparison. Data collection was conducted concurrently in Afikpo, Abakaliki and Ikwo axes. One trained researcher coordinated each axis, supported by research assistants. Identical data collection tools and water sampling protocols were used across all communities to ensure consistency and data quality.

### Study population

The study population comprised households living in selected flood-affected communities in Ebonyi State, Nigeria. Adult household heads or primary caregivers aged 18 years and above were interviewed as respondents. The analysis focused on households with at least one child under-five years of age, as the main outcome was diarrhoea among under-five children. A total of 600 households were included in the survey and one eligible adult respondent was interviewed in each household. Information collected covered household WASH conditions, flood-related disruption and the recent health status of the selected under-five child. Households and respondents were eligible if located in one of the selected flood-affected communities, 18 years or older, a permanent resident, had at least one child under-five years of age and provided informed consent. Households were excluded if the respondent was unable to provide reliable information, was severely ill at the time of data collection, declined to provide informed consent, or if the household did not have an eligible under-five child. These criteria ensured that respondents had sufficient knowledge of household WASH conditions and flooding effects.

### Sample size determination

The sample size was determined using Cochran’s formula for estimating a single population proportion [28], based on the primary outcome of under-five diarrhoea prevalence. As no local prevalence estimate was available, a prevalence of 50%, 95% confidence level and 5% margin of error were assumed.

$$\mathrm{n}\;=\;\mathrm{Z}^{2}\mathrm{pq}\;/\;\mathrm{d}^{2}$$

Where n is the minimum sample size, Z is the standard normal deviate, p is the estimated prevalence, q = 1 – p and d are the desired precision. The minimum sample size was 384 households. Applying a design effect of 1.5 increased this to 576 households. To allow for non-response and incomplete data, the target sample size was increased to 600 households. During data collection, 630 questionnaires were distributed (105 per community), of which 600 were completed and included in the analysis (response rate: 95.2%). One hundred households from each community were included in the final analysis.

### Sampling technique

A multistage sampling technique was used to select households for the quantitative survey. In the first stage, Ebonyi State was organized into three study axes based on the selected flood-affected communities: Afikpo, Abakaliki and Ikwo. Within each axis, communities with a history of recurrent flooding were identified using local records, field information and community reports. Based on these criteria, six communities were selected: Enohia Itim and Kpoghirikpo in Afikpo, Azuoto-Okpuitumo and Onakpoke in Abakaliki and Inyimagu and Igbudu in Ikwo. In the second stage, household listing was conducted in each community to identify eligible households. Only households with at least one child under-five years were included in the sampling frame. In the third stage, households were selected using systematic random sampling. One hundred households were chosen from each community, giving a total of 600 households. A sampling interval was calculated separately for each site. The first household was selected randomly and every ***kth*** household was subsequently chosen until the target number was reached. In each household, one adult respondent was interviewed.

### Data collection instruments and procedures

Data were collected using an interviewer-administered questionnaire developed specifically for this study (Additional File 1). The instrument was developed to obtain information on household WASH conditions, point-of-use drinking water contamination and diarrhoea among under-five children in the selected flood-affected communities. It covered socio-demographic characteristics, flood-related WASH disruption, water access, treatment and storage practices, sanitation facilities, hygiene practices and recent diarrhoea among under-five children. Data collection was conducted by trained field assistants under the supervision of one researcher in each study axis, with support from local guides when necessary. Face-to-face interviews were held with household heads or primary caregivers knowledgeable about household WASH practices and child health. Household observations and point-of-use drinking water samples were also collected following the study protocol to ensure consistency across communities.

### Quality assurance

The questionnaire was reviewed by public health experts from Alex Ekwueme Federal University, Ndufu-Alike, Nnamdi Azikiwe University, Awka and the Ebonyi State Ministry of Health to assess content validity, clarity and relevance. It was pretested in Atani, Ogbaru LGA, Anambra State and revised before data collection. Data collectors received training on study procedures, questionnaire administration and water sample collection. Completed questionnaires were checked for completeness. Standard procedures guided sampling, transport and laboratory analysis to ensure reliable results.

### Water sampling procedure

Drinking water samples were collected from all the 600 households included in the survey, with 100 samples obtained from each of the six study communities. Samples were taken from the main point-of-use container used for storing household drinking water. Stored water was selected because it reflects the quality of water actually consumed and captures contamination introduced during handling and storage. Sampling was conducted after completion of the household interview. Where a dispensing tap was available, water was collected directly into the sample bottle. Where no tap was present, water was transferred aseptically using a sterile pour method. Samples were collected in sterile 500 mL polypropylene bottles labelled with household identification number, date and time. Approximately 250 mL of water was collected per household. Immediately after collection, samples were placed in insulated cool boxes maintained at 4–8 °C and transported to the laboratory within four hours. All samples were processed on the same day following WHO and APHA standard microbiological procedures. Duplicate analysis was conducted on 10% of samples for quality control.

### Microbiological water analysis

Microbiological analysis was conducted in the Department of Microbiology, Ebonyi State University, under standard biosafety conditions. The membrane filtration method was used to detect *Escherichia coli* and total coliforms in household drinking water samples. For each sample, 100 mL of water was filtered through a 0.45 μm membrane filter using a vacuum filtration unit. The membrane was then placed on selective culture media. For *E. coli*, filters were incubated on m-FC agar at 44.5 °C for 24 h. For total coliforms, filters were incubated on m-Endo agar at 35 °C for 24 h. After incubation, colonies were counted and recorded as colony-forming units per 100 mL (CFU/100 mL). Blue colonies on m-FC agar were identified as *E. coli*, while pink to red colonies with metallic sheen on m-Endo agar were recorded as total coliforms. Contamination levels were classified according to WHO risk categories. Duplicate analysis and appropriate controls were included for quality assurance.

### Measurement of variables

The primary outcome variable was diarrhoea among under five children. Diarrhoea was defined as caregiver reported passage of three or more loose or watery stools within a 24-hour period during the two-weeks preceding the survey. It was recorded as a binary outcome (yes/no). The main exposure variables were flood related WASH disruption and point of use drinking water contamination. Flood related WASH disruption was measured using household indicators referring to the most recent flood event experienced by the household, including direct flood exposure, disrupted drinking water access, flooding of the main water source, flooding of toilet facilities, temporary displacement and stagnant water around the dwelling. Household WASH conditions included drinking water source, location, collection time, functionality, treatment, storage practices, toilet facilities, child faeces disposal and handwashing conditions. Two storage variables were assessed: storage container type (covered or uncovered) and whether the container was covered at the time of the household visit. Drinking water contamination was determined through microbiological analysis for *Escherichia coli* and total coliform. Contamination was classified as negative (0 CFU/100 mL) or positive (≥ 1 CFU/100 mL), with risk categories based on colony counts.

### Method of data analysis

Of the 630 households approached, 600 completed the questionnaire and were included in the analysis, giving a response rate of 95.2%. Data were analysed using SPSS version 25. Descriptive statistics were used to summarise respondent characteristics, household WASH conditions, water contamination and diarrhoea prevalence. Associations between categorical variables and under-five diarrhoea were examined using the chi-square test. Variables associated with diarrhoea in the bivariate analysis, together with those considered epidemiologically relevant based on prior evidence, were entered into a multivariable logistic regression model. Multicollinearity was assessed using variance inflation factors (VIF) and tolerance statistics, with no evidence of problematic multicollinearity. Model fit was assessed using the Omnibus and Hosmer–Lemeshow tests. The outcome was household diarrhoea in at least one under-five child during the 14 days preceding the survey. Results are presented as adjusted odds ratios (AORs) with 95% confidence intervals. Statistical significance was set at *p* < 0.05. Laboratory results were expressed as CFU/100 mL and classified using WHO risk categories.

## Results

Of the 630 households approached, 600 completed the questionnaire and were included in the analysis, giving a response rate of 95.2%.

Table 1 presents the sociodemographic and household characteristics of the study population by diarrhoea status. Among the 600 respondents, slightly more than half were female (54.2%) and the largest age group was 18–29 years (30.0%). Most caregivers were mothers (58.5%), and farming was the predominant occupation (35.3%). Households commonly comprised five to six members (33.7%) and about half had one under-five child (50.7%). Mud or thatched structures were the most frequent dwelling type (31.2%), while zinc or iron sheets were the most common roofing materials (59.0%). None of the sociodemographic variables showed a statistically significant association with diarrhoea (all *p* > 0.05), suggesting that diarrhoeal episodes were not concentrated within any particular demographic subgroup.

**Table 1:** Sociodemographic and household characteristics of study participants by diarrhoea status (*n* = 600)

Variable	Category	Total *n* (%)	No diarrhoea *n* = 460 (%)	Diarrhoea *n* = 140 (%)	χ²	df	*p*-value
Sex	Male	275 (45.8)	210 (45.7)	65 (46.4)	0.026	1	0.872
	Female	325 (54.2)	250 (54.3)	75 (53.6)			
Age (years)	18–29	180 (30.0)	142 (30.9)	38 (27.1)	2.912	4	0.573
	30–39	168 (28.0)	126 (27.4)	42 (30.0)			
	40–49	124 (20.7)	99 (21.5)	25 (17.9)			
	50–59	72 (12.0)	51 (11.1)	21 (15.0)			
	≥ 60	56 (9.3)	42 (9.1)	14 (10.0)			
Relationship	Mother	351 (58.5)	271 (58.9)	80 (57.1)	3.874	4	0.423
	Father	134 (22.3)	106 (23.0)	28 (20.0)			
	Grandparent	64 (10.7)	43 (9.3)	21 (15.0)			
	Other caregiver	25 (4.2)	20 (4.3)	5 (3.6)			
	Household head	26 (4.3)	20 (4.3)	6 (4.3)			
Education	No formal	92 (15.3)	69 (15.0)	23 (16.4)	6.499	4	0.165
	Primary	151 (25.2)	111 (24.1)	40 (28.6)			
	Secondary	194 (32.3)	159 (34.6)	35 (25.0)			
	Tertiary	119 (19.8)	85 (18.5)	34 (24.3)			
	Other	44 (7.3)	36 (7.8)	8 (5.7)			
Occupation	Farming	212 (35.3)	162 (35.2)	50 (35.7)	3.831	5	0.574
	Trading	144 (24.0)	109 (23.7)	35 (25.0)			
	Civil service	76 (12.7)	60 (13.0)	16 (11.4)			
	Artisan	90 (15.0)	64 (13.9)	26 (18.6)			
	Unemployed	52 (8.7)	43 (9.3)	9 (6.4)			
	Other	26 (4.3)	22 (4.8)	4 (2.9)			
Household size	1–2	66 (11.0)	51 (11.1)	15 (10.7)	2.973	3	0.396
	3–4	189 (31.5)	137 (29.8)	52 (37.1)			
	5–6	202 (33.7)	161 (35.0)	41 (29.3)			
	≥ 7	143 (23.8)	111 (24.1)	32 (22.9)			
Under-5 children	1	304 (50.7)	230 (50.0)	74 (52.9)	1.127	3	0.771
	2	179 (29.8)	136 (29.6)	43 (30.7)			
	3	88 (14.7)	71 (15.4)	17 (12.1)			
	≥ 4	29 (4.8)	23 (5.0)	6 (4.3)			
Dwelling type	Temporary	121 (20.2)	91 (19.8)	30 (21.4)	7.497	4	0.112
	Mud/thatched	187 (31.2)	143 (31.1)	44 (31.4)			
	Block/brick	142 (23.7)	103 (22.4)	39 (27.9)			
	Mixed	116 (19.3)	91 (19.8)	25 (17.9)			
	Other	34 (5.7)	32 (7.0)	2 (1.4)			
Roofing material	Thatch/grass	123 (20.5)	99 (21.5)	24 (17.1)	2.054	3	0.561
	Zinc/iron sheet	354 (59.0)	265 (57.6)	89 (63.6)			
	Concrete/tile	86 (14.3)	66 (14.3)	20 (14.3)			
	Other	37 (6.2)	30 (6.5)	7 (5.0)			

Data are n (%). χ² = chi-square test. No sociodemographic variable was significantly associated with diarrhoea (all *p* > 0.05). Significance set at *p* < 0.05

Table 2 shows the extent of flood-related disruption and its relationship with under-five diarrhoea. Most households (69.5%) were directly affected by flooding, with moderate impact reported most frequently (44.2%). Over half experienced disrupted water access (59.7%) and 43.3% reported partial damage to their main drinking water source. Stagnant water was observed around two-thirds of dwellings (66.2%) and nearly half of households (49.3%) were temporarily displaced. Among displaced households, staying with relatives or friends was most common (30.5%) and displacement typically lasted 6–10 days (29.5%). None of the flood-related factors was significantly associated with diarrhoea (all *p* > 0.05). Only visible stagnant water showed a marginal association with diarrhoea at the 10% significance level (*p* = 0.078), whereas household displacement was not significantly associated (*p* = 0.181).

**Table 2:** Flood-related disruption and association with under-five diarrhoea (*n* = 600)

Variable	Category	Total *n* (%)	No diarrhoea *n* = 460 (%)	Diarrhoea *n* = 140 (%)	χ²	df	*p*-value
Flood-affected	No	183 (30.5)	142 (30.9)	41 (29.3)	0.127	1	0.722
	Yes	417 (69.5)	318 (69.1)	99 (70.7)			
Severity	Mild	180 (30.0)	138 (30.0)	42 (30.0)	0.573	2	0.751
	Moderate	265 (44.2)	200 (43.5)	65 (46.4)			
	Severe	155 (25.8)	122 (26.5)	33 (23.6)			
Water source affected	No	218 (36.3)	168 (36.5)	50 (35.7)	0.067	2	0.967
	Partially	260 (43.3)	198 (43.0)	62 (44.3)			
	Severely	122 (20.3)	94 (20.4)	28 (20.0)			
Toilet affected	No	253 (42.2)	197 (42.8)	56 (40.0)	3.591	3	0.309
	Partially	181 (30.2)	131 (28.5)	50 (35.7)			
	Severely	118 (19.7)	96 (20.9)	22 (15.7)			
	No toilet before	48 (8.0)	36 (7.8)	12 (8.6)			
Water access disrupted	No	242 (40.3)	189 (41.1)	53 (37.9)	0.465	1	0.495
	Yes	358 (59.7)	271 (58.9)	87 (62.1)			
Stagnant water	No	203 (33.8)	147 (32.0)	56 (40.0)	3.102	1	0.078
	Yes	397 (66.2)	313 (68.0)	84 (60.0)			
Displaced	No	304 (50.7)	240 (52.2)	64 (45.7)	1.792	1	0.181
	Yes	296 (49.3)	220 (47.8)	76 (54.3)			
Displacement location	Camp/shelter	141 (23.5)	109 (23.7)	32 (22.9)	1.670	5	0.893
	School building	118 (19.7)	86 (18.7)	32 (22.9)			
	Relative/friend	183 (30.5)	141 (30.7)	42 (30.0)			
	Temporary structure	95 (15.8)	73 (15.9)	22 (15.7)			
	Other	30 (5.0)	24 (5.2)	6 (4.3)			
	Not applicable	33 (5.5)	27 (5.9)	6 (4.3)			
Displacement duration	1–5 days	167 (27.8)	125 (27.2)	42 (30.0)	1.004	3	0.800
	6–10 days	177 (29.5)	134 (29.1)	43 (30.7)			
	11–20 days	153 (25.5)	119 (25.9)	34 (24.3)			
	≥ 21 days	103 (17.2)	82 (17.8)	21 (15.0)			

Data are n (%). χ² = chi-square test. No flood-related variable was statistically significant at the 5% level. Visible stagnant water showed a marginal association at the 10% significance level (*p* = 0.078), whereas household displacement was not associated

**Fig. 2:**
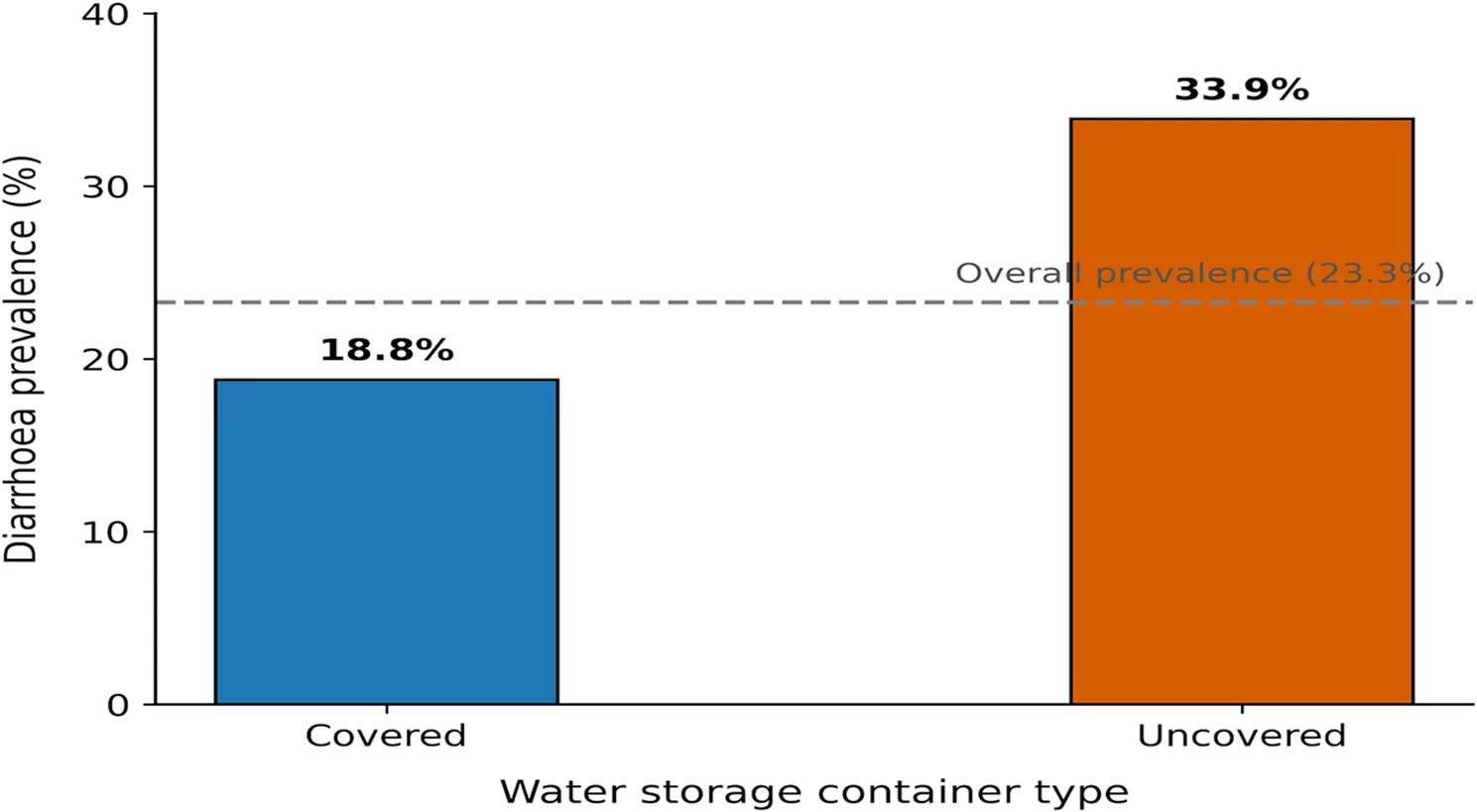
Under-five diarrhoea prevalence by water storage container type in flood-displaced communities in Ebonyi State, Nigeria. Households using uncovered water storage containers had a significantly higher prevalence of diarrhoea than those using covered containers (χ² = 14.420, *p* < 0.001). The dashed horizontal line indicates the overall diarrhoea prevalence of 23.3%

Table 3 outlines household WASH conditions and their relationship with under-five diarrhoea. Piped water or borehole was the most common drinking water source (41.7%) and 51.8% of households reported that their water source was always functional. Most households (60.3%) did not treat drinking water before use, while 70.0% stored water in covered containers. Shared toilet facilities were used by 61.5% of households and 36.0% disposed of child faeces in a toilet or latrine. Handwashing facilities and soap were available in 73.3% and 72.8% of households, respectively. Water treatment method (*p* = 0.020) and water storage container type (*p* < 0.001) were significantly associated with under-five diarrhoea. Households using uncovered water storage containers had a higher prevalence of diarrhoea than those using covered containers (Fig. 2). Household water treatment was not significantly associated with diarrhoea (*p* = 0.095). No significant associations were observed for water source, toilet type, shared toilet use, child faeces disposal, or hand hygiene practices (all *p* > 0.05).

**Table 3:** Household WASH conditions and association with under-five diarrhoea (*n* = 600)

Variable	Category	Total *n* (%)	No diarrhoea *n* (%)	Diarrhoea *n* (%)	χ²	df	*p*-value
Main water source	Piped/Borehole	250 (41.7)	201 (80.4)	49 (19.6)	8.360	7	0.399
	Protected well	84 (14.0)	69 (82.1)	15 (17.9)			
	Unprotected well	51 (8.5)	38 (74.5)	13 (25.5)			
	Rainwater	26 (4.3)	18 (69.2)	8 (30.8)			
	River/stream	85 (14.2)	60 (70.6)	25 (29.4)			
	Tanker/vendor	37 (6.2)	26 (70.3)	11 (29.7)			
	Sachet/bottled	50 (8.3)	36 (72.0)	14 (28.0)			
	Other	17 (2.8)	12 (70.6)	5 (29.4)			
Water source functional	Always	311 (51.8)	244 (78.5)	67 (21.5)	1.820	2	0.403
	Sometimes	195 (32.5)	143 (73.3)	52 (26.7)			
	Rarely	94 (15.7)	73 (77.7)	21 (22.3)			
Treats drinking water before use	No	362 (60.3)	286 (79.0)	76 (21.0)	2.791	1	0.095
	Yes	238 (39.7)	174 (73.1)	64 (26.9)			
Water treatment method	No treatment	360 (60.0)	289 (80.3)	71 (19.7)	5.380	1	0.020
	Any treatment	240 (40.0)	171 (71.3)	69 (28.7)			
Water storage container type	Covered	420 (70.0)	341 (81.2)	79 (18.8)	14.420	1	< 0.001
	Uncovered	180 (30.0)	119 (66.1)	61 (33.9)			
Toilet type	Flush toilet	58 (9.7)	41 (70.7)	17 (29.3)	8.341	5	0.303
	VIP latrine	85 (14.2)	64 (75.3)	21 (24.7)			
	Pit latrine (slab)	124 (20.7)	87 (70.2)	37 (29.8)			
	Pit latrine (no slab)	133 (22.2)	108 (81.2)	25 (18.8)			
	No facility	110 (18.3)	88 (80.0)	22 (20.0)			
	Other	90 (15.0)	72 (80.0)	18 (20.0)			
Shared toilet	No	231 (38.5)	177 (76.6)	54 (23.4)	0.000	1	0.984
	Yes	369 (61.5)	283 (76.7)	86 (23.3)			
Child faeces disposal	Toilet/latrine	216 (36.0)	157 (72.7)	59 (27.3)	7.926	6	0.244
	Buried	83 (13.8)	61 (73.5)	22 (26.5)			
	Refuse dump	85 (14.2)	63 (74.1)	22 (25.9)			
	Open ground	92 (15.3)	76 (82.6)	16 (17.4)			
	Drain/water body	64 (10.7)	52 (81.3)	12 (18.7)			
	Child uses toilet	33 (5.5)	29 (87.9)	4 (12.1)			
	Other	27 (4.5)	22 (81.5)	5 (18.5)			
Handwashing place	No	160 (26.7)	123 (76.9)	37 (23.1)	0.005	1	0.942
	Yes	440 (73.3)	337 (76.6)	103 (23.4)			
Soap available	No	163 (27.2)	120 (73.6)	43 (26.4)	1.162	1	0.281
	Yes	437 (72.8)	340 (77.8)	97 (22.2)			
Handwashing after defecation	No	159 (26.5)	121 (76.1)	38 (23.9)	0.039	1	0.844
	Yes	441 (73.5)	339 (76.9)	102 (23.1)			

Data are presented as n (%). Percentages for the “No diarrhoea” and “Diarrhoea” columns are row percentages (i.e., percentages within each category). χ² = Pearson chi-square test. Statistical significance was set at *p* < 0.05. Water treatment method (*p* = 0.020) and water storage container type (*p* < 0.001) were significantly associated with under-five diarrhoea

**Fig. 3:**
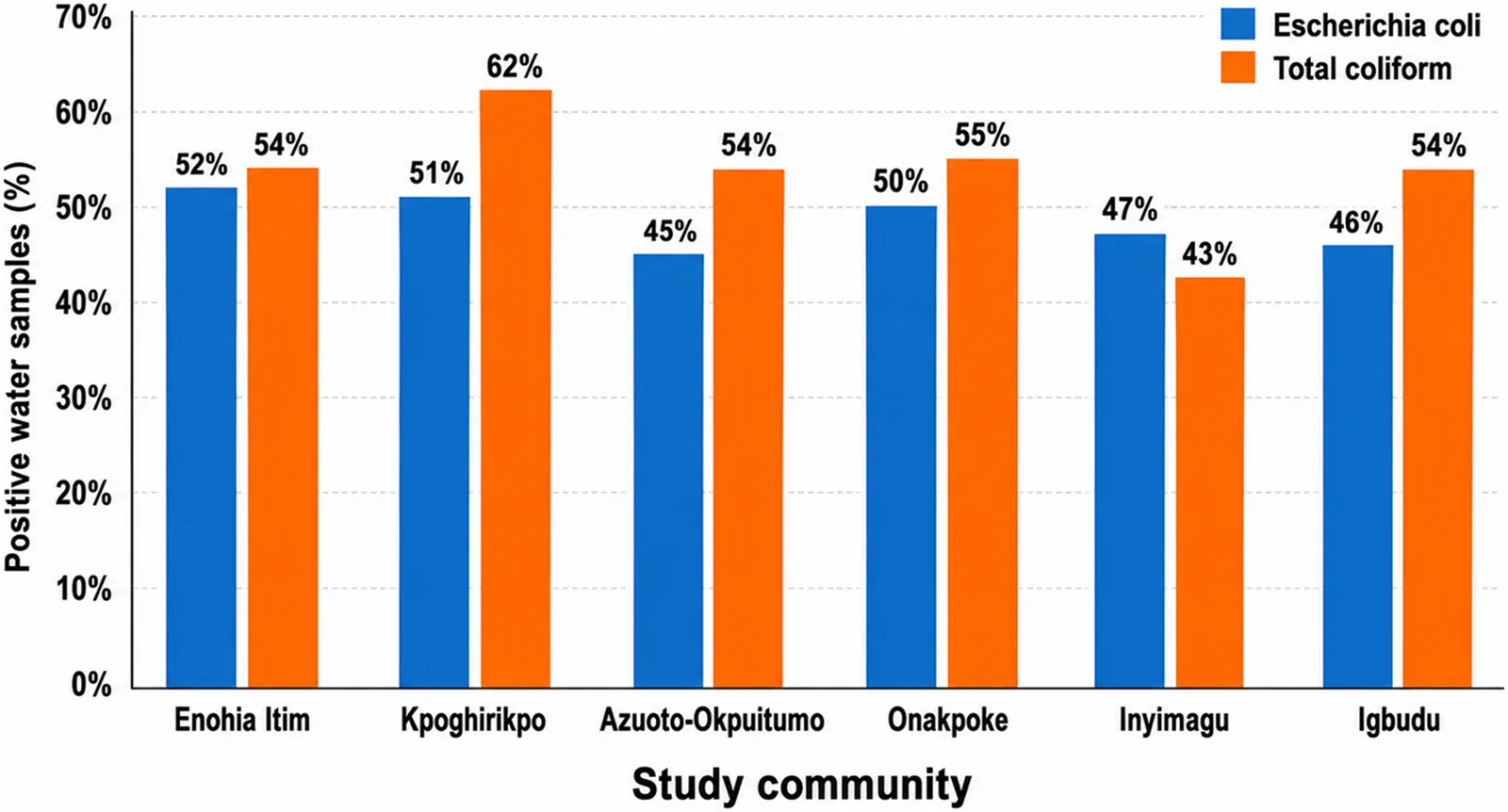
Percentage of *Escherichia coli*- and total coliform-positive drinking water samples across the six flood-displaced communities in Ebonyi State, Nigeria. No statistically significant differences were observed among communities for *Escherichia coli* contamination (χ² = 4.126, *p* = 0.532) or total coliform contamination (χ² = 7.284, *p* = 0.201)

Table 4; Fig. 3 present the microbiological quality of household drinking water across the six flood-displaced communities. Figure 3 illustrates the percentage of *Escherichia coli*-positive and total coliform-positive drinking water samples in each community. Overall, 291 (48.5%) water samples tested positive for *Escherichia coli*, while 322 (53.7%) tested positive for total coliforms. Among all samples, 24.5% were classified as medium risk (1–10 CFU/100 mL), 13.8% as high risk (11–100 CFU/100 mL) and 10.2% as very high risk (> 100 CFU/100 mL) for *Escherichia coli* contamination. Across the six communities, *Escherichia coli* positivity ranged from 45.0% in Azuoto-Okpuitumo to 52.0% in Enohia Itim, whereas total coliform positivity ranged from 43.0% in Inyimagu to 62.0% in Kpoghirikpo. The highest proportion of very high-risk *Escherichia coli* contamination was observed in Onakpoke (13.0%), while the lowest was observed in Igbudu (6.0%). No statistically significant differences were observed among communities in *Escherichia coli* contamination (χ² = 4.126, *p* = 0.532), total coliform contamination (χ² = 7.284, *p* = 0.201), *Escherichia coli* risk categories (χ² = 8.941, *p* = 0.442), or total coliform risk categories (χ² = 11.376, *p* = 0.328), indicating that microbial contamination was widespread across the study communities.

**Table 4:** Point-of-use drinking water contamination by study community (*N* = 600). Overall indicator

Indicator	*n* (%)							
*E. coli* positive	291 (48.5)							
Total coliform positive	322 (53.7)							

Variable	Eno *n* = 100 (%)	Kpo *n* = 100 (%)	Azu *n* = 100 (%)	Ona *n* = 100 (%)	Iny *n* = 100 (%)	Igb *n* = 100 (%)	χ²	*p*-value
E. coli contamination							**4.126**	**0.532**
Negative	48 (48.0)	49 (49.0)	55 (55.0)	50 (50.0)	53 (53.0)	54 (54.0)		
Positive	52 (52.0)	51 (51.0)	45 (45.0)	50 (50.0)	47 (47.0)	46 (46.0)		
Total coliform contamination							**7.284**	**0.201**
Negative	46 (46.0)	38 (38.0)	46 (46.0)	45 (45.0)	57 (57.0)	46 (46.0)		
Positive	54 (54.0)	62 (62.0)	54 (54.0)	55 (55.0)	43 (43.0)	54 (54.0)		
E. coli risk category							**8.941**	**0.442**
0 CFU/100 mL	48 (48.0)	49 (49.0)	55 (55.0)	50 (50.0)	53 (53.0)	54 (54.0)		
1–10 CFU/100 mL	29 (29.0)	25 (25.0)	24 (24.0)	22 (22.0)	22 (22.0)	25 (25.0)		
11–100 CFU/100 mL	12 (12.0)	17 (17.0)	10 (10.0)	15 (15.0)	14 (14.0)	15 (15.0)		
>100 CFU/100 mL	11 (11.0)	9 (9.0)	11 (11.0)	13 (13.0)	11 (11.0)	6 (6.0)		
Total coliform risk category							**11.376**	**0.328**
0 CFU/100 mL	46 (46.0)	38 (38.0)	46 (46.0)	45 (45.0)	57 (57.0)	46 (46.0)		
1–10 CFU/100 mL	34 (34.0)	30 (30.0)	30 (30.0)	26 (26.0)	25 (25.0)	34 (34.0)		
11–100 CFU/100 mL	11 (11.0)	19 (19.0)	10 (10.0)	18 (18.0)	10 (10.0)	12 (12.0)		
>100 CFU/100 mL	9 (9.0)	13 (13.0)	14 (14.0)	11 (11.0)	8 (8.0)	8 (8.0)		

Data are presented as n (%), with percentages calculated within each community (*n* = 100). χ² = Pearson chi-square test. *Eno* Enohia Itim, *Kpo* Kpoghirikpo, *Azu* Azuoto-Okpuitumo, *Ona* Onakpoke, *Iny* Inyimagu, *Igb* Igbudu. WHO risk categories were defined as low (0 CFU/100 mL), medium (1–10 CFU/100 mL), high (11–100 CFU/100 mL) and very high (> 100 CFU/100 mL). CFU = colony-forming units per 100 mL. No statistically significant differences in *Escherichia coli* contamination, total coliform contamination, or contamination risk categories were observed across the six communities (all *p* > 0.05)

Table 5 examines the relationship between point-of-use drinking water contamination and under-five diarrhoea. *E. coli* contamination was more common among children with diarrhoea (52.9%) than among those without diarrhoea (45.0%), although the difference was not statistically significant (χ² = 2.661, *p* = 0.103). A similar pattern was observed for total coliform contamination, which was higher in the diarrhoea group (62.9%) compared to the non-diarrhoea group (56.1%) (χ² = 2.015, *p* = 0.156). No significant differences were found across *E. coli* risk categories (*p* = 0.610) or total coliform risk categories (*p* = 0.896). While contamination levels tended to be higher in households reporting diarrhoea, these associations did not reach statistical significance.

**Table 5:** Association between point-of-use drinking water contamination and under-five diarrhoea (*n* = 600)

Variable	No Diarrhoea *n* = 460 (%)	Diarrhoea *n* = 140 (%)	χ²	*p*-value
*E. coli* contamination			2.661	0.103
Negative	253 (55.0)	66 (47.1)		
Positive	207 (45.0)	74 (52.9)		
Total coliform contamination			2.015	0.156
Negative	202 (43.9)	52 (37.1)		
Positive	258 (56.1)	88 (62.9)		
*E. coli* risk category			1.825	0.610
0 CFU/100mL	235 (51.1)	74 (52.9)		
1–10 CFU/100mL	111 (24.1)	37 (26.4)		
11–100 CFU/100mL	66 (14.3)	14 (10.0)		
> 100 CFU/100mL	48 (10.4)	15 (10.7)		
Total coliform risk category			0.600	0.896
0 CFU/100mL	213 (46.3)	65 (46.4)		
1–10 CFU/100mL	137 (29.8)	43 (30.7)		
11–100 CFU/100mL	63 (13.7)	16 (11.4)		
> 100 CFU/100mL	47 (10.2)	16 (11.4)		

*E. coli* positivity was higher among children with diarrhoea (52.9%) than those without (45.0%), though the difference was not statistically significant (χ² = 2.661, *p* = 0.103). Total coliform positivity was similarly higher in the diarrhoea group (62.9% vs. 56.1%; χ² = 2.015, *p* = 0.156). These findings suggest a consistent trend of higher contamination in households with diarrhoea cases, though statistical significance was not reached

Table 6 presents the association between household water practices and *Escherichia coli* contamination of point-of-use drinking water. Water treatment method was significantly associated with *Escherichia coli* contamination (χ² = 5.741, *p* = 0.023). Although most households (60.3%) did not treat their drinking water before use, households using different treatment methods showed varying levels of *Escherichia coli* contamination. Water sources located outside the household compound showed a marginal association with *Escherichia coli* contamination at the 10% significance level (χ² = 3.749, *p* = 0.053). Other factors, including the main water source, storage container type and whether the storage container was covered at the time of the household visit, were not significantly associated with *Escherichia coli* contamination (all *p* > 0.05).

**Table 6:** Bivariate association between household water practices and *E. coli* contamination (*n* = 600)

Variable	E. coli Negative *n* = 319 (%)	E. coli Positive *n* = 281 (%)	χ²	*p*-value
Main water source			10.353	0.241
Piped water	37 (11.6)	24 (8.5)		
Borehole	103 (32.3)	86 (30.6)		
Protected well	52 (16.3)	32 (11.4)		
Unprotected well	27 (8.5)	24 (8.5)		
Rainwater	15 (4.7)	11 (3.9)		
River/stream	36 (11.3)	49 (17.4)		
Tanker/vendor	19 (6.0)	18 (6.4)		
Sachet/bottled	23 (7.2)	27 (9.6)		
Other	7 (2.2)	10 (3.6)		
Water source within compound			3.749	**0.053**
No	161 (50.5)	164 (58.4)		
Yes	158 (49.5)	117 (41.6)		
Water source functionality			3.650	0.161
Always functional	169 (53.0)	142 (50.5)		
Sometimes functional	94 (29.5)	101 (35.9)		
Rarely functional	56 (17.6)	38 (13.5)		
Treats water before use			0.335	0.562
No	189 (59.2)	173 (61.6)		
Yes	130 (40.8)	108 (38.4)		
Treatment method			5.741	0.023*
None	191 (59.9)	176 (62.6)		
Boiling	40 (12.5)	34 (12.1)		
Chlorination	20 (6.3)	21 (7.5)		
Filtration	13 (4.1)	13 (4.6)		
Sedimentation	18 (5.6)	19 (6.8)		
Cloth straining	18 (5.6)	7 (2.5)		
Other	19 (6.0)	11 (3.9)		
Storage container type			0.619	0.961
Covered bucket with lid	125 (39.2)	113 (40.2)		
Open bucket	49 (15.4)	41 (14.6)		
Jerry can	81 (25.4)	73 (26.0)		
Clay pot	34 (10.7)	32 (11.4)		
Other	30 (9.4)	22 (7.8)		
Container covered during household visit			1.699	0.192
No	91 (28.5)	94 (33.5)		
Yes	228 (71.5)	187 (66.5)		

Data are presented as n (%). Pearson’s chi-square test was used for comparisons. Escherichia coli contamination was defined as ≥ 1 CFU/100 mL. Treatment method was significantly associated with Escherichia coli contamination (χ² = 5.741, *p* = 0.023). Water source within the compound showed a marginal association with Escherichia coli contamination at the 10% significance level (χ² = 3.749, *p* = 0.053). Statistical significance was defined as *p* < 0.05

**Fig. 4:**
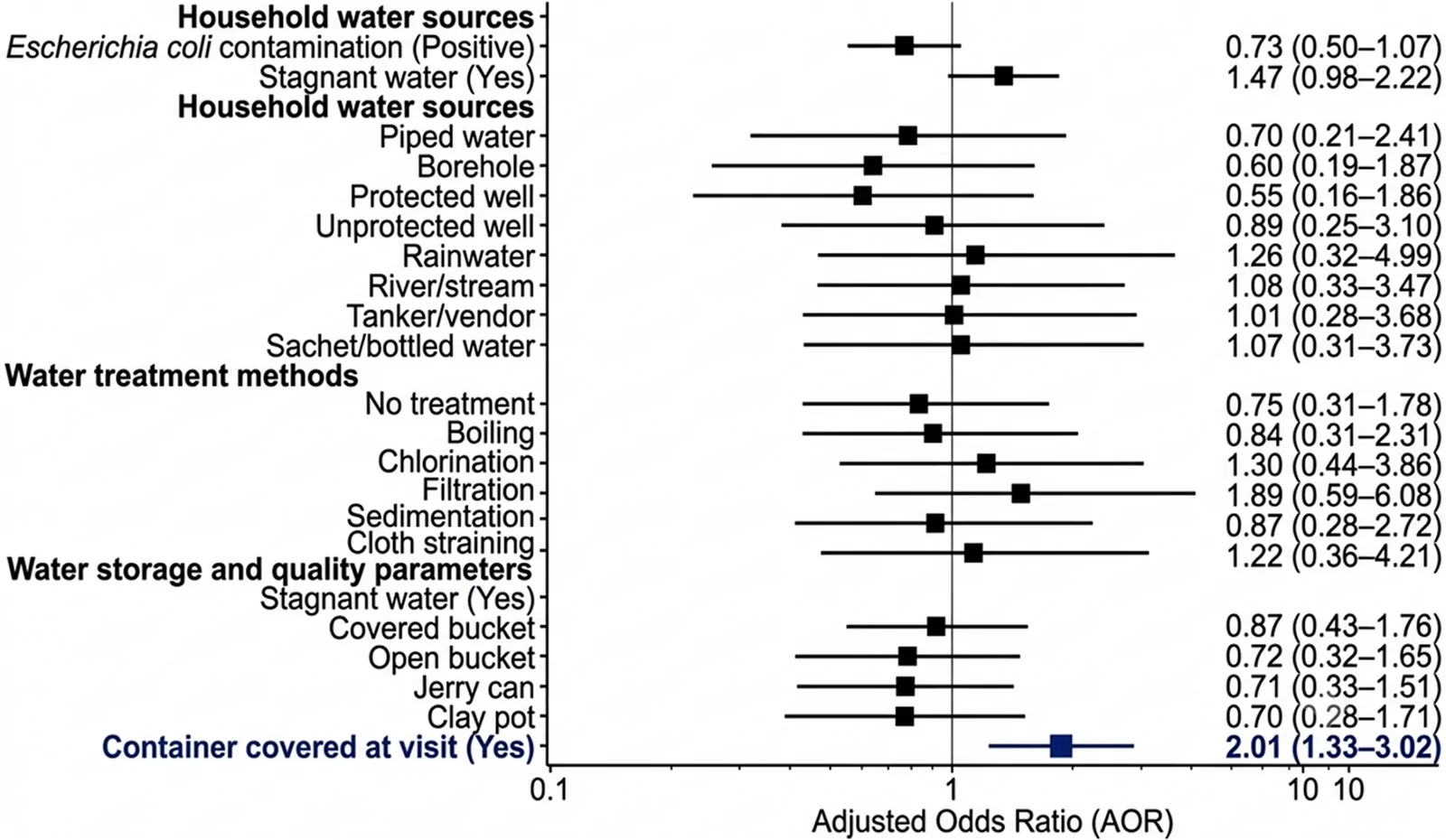
Forest plot of adjusted odds ratios (AORs) and 95% confidence intervals for predictors of under-five diarrhoea in flood-displaced communities in Ebonyi State, Nigeria. The vertical reference line indicates AOR = 1.0. The only statistically significant association was observed for a storage container observed to be covered during the household visit (AOR = 2.01, 95% CI: 1.33–3.02). This finding should be interpreted cautiously because reverse causation and residual confounding cannot be excluded

Table 7 presents the multivariable logistic regression analysis of predictors of under-five diarrhoea. The overall model was statistically significant (Omnibus χ² = 31.525, df = 20, *p* = 0.049) and showed good fit based on the Hosmer–Lemeshow test (χ² = 5.713, df = 8, *p* = 0.679). No evidence of problematic multicollinearity was identified (VIF: 1.002–1.017). The model explained 7.7% of the variation in diarrhoea (Nagelkerke R² = 0.077), with an overall classification accuracy of 75.8%. After adjustment, *Escherichia coli* contamination was not significantly associated with diarrhoea (AOR = 0.73, 95% CI: 0.50–1.07, *p* = 0.103). Stagnant water around the dwelling was not significantly associated with diarrhoea (AOR = 1.47, 95% CI: 0.98–2.22, *p* = 0.063), although a possible trend was observed. Main water source, treatment method and storage container type were also not independently associated with diarrhoea (all *p* > 0.05). After adjustment for the variables included in the model, a storage container observed to be covered during the household visit remained significantly associated with diarrhoea (AOR = 2.01, 95% CI: 1.33–3.02, *p* = 0.001). In the bivariate analysis, however, households with covered storage containers had a lower prevalence of diarrhoea (Fig. 4).

**Table 7:** Multivariable logistic regression analysis of predictors of under-five diarrhoea (*n* = 600)

Variable	Category	AOR (95% CI)	*p*-value
*E. coli* contamination	Negative	1 (ref)	—
	Positive	0.73 (0.50–1.07)	0.103
Stagnant water	No	1 (ref)	—
	Yes	1.47 (0.98–2.22)	0.063
Main water source	Other	1 (ref)	—
	Piped water	0.70 (0.21–2.41)	0.575
	Borehole	0.60 (0.19–1.87)	0.379
	Protected well	0.55 (0.16–1.86)	0.336
	Unprotected well	0.89 (0.25–3.10)	0.848
	Rainwater	1.26 (0.32–4.99)	0.739
	River/stream	1.08 (0.33–3.47)	0.903
	Tanker/vendor	1.01 (0.28–3.68)	0.988
	Sachet/bottled	1.07 (0.31–3.73)	0.919
Treatment method	Other	1 (ref)	—
	None	0.75 (0.31–1.78)	0.508
	Boiling	0.84 (0.31–2.31)	0.737
	Chlorination	1.30 (0.44–3.86)	0.632
	Filtration	1.89 (0.59–6.08)	0.287
	Sedimentation	0.87 (0.28–2.72)	0.808
	Cloth straining	1.22 (0.36–4.21)	0.748
Storage container	Other	1 (ref)	—
	Covered bucket	0.87 (0.43–1.76)	0.694
	Open bucket	0.72 (0.32–1.65)	0.441
	Jerry can	0.71 (0.33–1.51)	0.369
	Clay pot	0.70 (0.28–1.71)	0.429
Container observed to be covered during household visit†	No	1 (ref)	—
	Yes	**2.01 (1.33–3.02)**	**0.001**

*AOR* adjusted odds ratio, *CI* confidence interval. Variables were entered simultaneously using the Enter method with indicator contrast coding. Model fit: Omnibus χ² = 31.525, df = 20, *p* = 0.049; Hosmer–Lemeshow χ² = 5.713, df = 8, *p* = 0.679; Nagelkerke R² = 0.077; overall classification accuracy = 75.8%. Reference categories are indicated as ref. †This unexpected association should be interpreted cautiously because reverse causation and residual confounding cannot be excluded. Statistical significance was set at *p* < 0.05

## Discussion

This study examined flood-related WASH disruption, point-of-use drinking water contamination and diarrhoea among under-five children in flood-displaced communities in Ebonyi State, Nigeria. Three findings stand out. First, flood-related WASH disruption was widespread. Second, microbial contamination of household drinking water was high across all study communities. Third, under-five diarrhoea remained common. Nearly half of household water samples were positive for *Escherichia coli* (48.5%) and 53.7% contained total coliforms. The two-week diarrhoea prevalence was 23.3%. Sociodemographic factors were not associated with diarrhoea, suggesting that risk was more strongly influenced by environmental and household WASH conditions than demographic characteristics. The strongest association in the multivariable model involved household water storage.

Flood-related disruption was extensive across the study communities. Most households reported direct flood exposure and disrupted drinking water access affected almost three-fifths of households. Stagnant water was also common around dwellings. This pattern is consistent with reports from flood-prone settings in sub-Saharan Africa, where flooding damages water and sanitation infrastructure [7, 8, 29]. In Ebonyi State, recurrent flooding has repeatedly disrupted infrastructure and displaced vulnerable populations [20]. The high level of reported flood exposure confirms that the study population experienced conditions likely to affect WASH access and disease transmission.

Although flood exposure was widespread, no flood-related variable was independently associated with diarrhoea. Flooding may influence diarrhoeal risk indirectly through effects on water access, sanitation, hygiene, shelter quality and environmental contamination. Only stagnant water showed a marginal association with diarrhoea (*p* = 0.078), whereas household displacement was not associated. Similar findings have been reported elsewhere. Yazdi et al. [8] found that flooding increased infectious diarrhoea risk, particularly where sanitation systems were weak, while Saha et al. [30] reported that this relationship was partly mediated by contaminated water and poor sanitation. The lack of statistical significance may also reflect the cross-sectional design, which captures a single point in time and cannot assess cumulative or delayed effects of flood exposure [5].

The high level of microbial contamination observed is a public health concern. Detection of *Escherichia coli* in 48.5% of household water samples and total coliforms in 53.7% indicates widespread faecal contamination and poor household water safety. Similar findings have been reported in other low-resource flood-affected settings. Niven et al. [31] linked water supply interruptions in peri-urban Malawi with deterioration in drinking water quality and child health, while Malebatja and Mokgatle [21] reported similar findings in South Africa. Contamination remained consistently high across all six communities, suggesting a systemic rather than localised problem.

Water treatment method was significantly associated with *Escherichia coli* contamination. Most households did not treat drinking water. Although cloth straining showed the lowest contamination, this may reflect better overall hygiene practices rather than the effectiveness of cloth straining itself. Households practising any form of water treatment may also be more likely to store and handle water safely. This interpretation agrees with previous studies showing that household water treatment and improved WASH practices reduce enteric infections [16, 32]. Water sources located outside the household compound also showed a marginal association with *Escherichia coli* contamination, consistent with contamination during collection, transport and storage [33].

The diarrhoea prevalence of 23.3% indicates a substantial disease burden in this flood-displaced population. Similar estimates have been reported among displaced populations and children living in poor WASH environments [4, 9]. The absence of associations with sociodemographic characteristics suggests that children shared common environmental exposures. Comparable findings have been reported in Madagascar and Pakistan, where household WASH conditions explained more variation in diarrhoeal disease than socioeconomic factors [6, 34].

The multivariable analysis identified a storage container observed to be covered during the household visit as the only significant predictor of diarrhoea. The adjusted association differed from the crude relationship observed in the bivariate analysis, in which households with covered storage containers had a lower prevalence of diarrhoea. This reversal may reflect residual confounding, complex interrelationships among household WASH characteristics, or reverse causation. For example, households experiencing recent diarrhoeal episodes may have adopted covering of water containers following illness or health education. In addition, contamination may have occurred before household storage or through inadequate cleaning of storage containers, deterioration in water quality during storage, and unsafe water handling practices [27, 35–37]. Because this study was cross-sectional, the temporal sequence between water storage practices and diarrhoeal episodes cannot be established. Consequently, this finding should not be interpreted as evidence that covering water containers increases diarrhoeal risk.

*Escherichia coli* contamination was not independently associated with diarrhoea after adjustment. This may reflect multiple transmission pathways, including person-to-person spread, foodborne exposure and environmental contamination [5]. In addition, the two-week recall period for diarrhoea may not correspond with water quality measured at the survey, resulting in exposure misclassification.

These findings highlight the importance of improving point-of-use water quality in flood-displaced communities. Emergency WASH interventions should strengthen household water treatment, safe storage, sanitation and environmental hygiene [32, 38]. A strength of this study is the integration of household survey data with microbiological water testing in an understudied population. However, the cross-sectional design, caregiver recall and single-time-point water sampling limit causal inference and generalisability. Multiple statistical comparisons were also performed, increasing the possibility of type I error. Despite these limitations, the findings provide useful evidence to strengthen flood-response WASH programmes in Nigeria.

### Summary of key findings

This study documented widespread flood-related WASH disruption, high microbial water contamination and elevated diarrhoea burden among under-five children in flood-displaced communities in Ebonyi State, Nigeria. Most households reported direct flood exposure, disrupted water access and stagnant water around their dwellings. Nearly half of water samples were positive for *Escherichia coli* (48.5%) and 53.7% contained total coliforms, far exceeding WHO safe water standards [1, 10]. Contamination was uniformly high across all six communities, reflecting a systemic water safety failure rather than a localised problem [24, 26]. The two-week diarrhoea prevalence was 23.3%. Sociodemographic factors were not associated with diarrhoea [6, 34]. Water treatment method was significantly associated with *Escherichia coli* contamination, consistent with previous evidence [16] and a storage container observed to be covered during the household visit was the only independent predictor of diarrhoea in the adjusted analysis.

### Strengths

A major strength of this study is the integration of household survey data with microbiological testing of stored drinking water. This approach provided both behavioural and objective evidence of WASH-related risk. Another strength is the focus on flood-displaced communities, an underrepresented population in WASH research despite documented vulnerability to diarrhoeal disease in humanitarian and climate-affected settings.

### Study limitations

This study has several limitations. The cross-sectional design precludes causal inference. Diarrhoea was based on caregiver recall over the preceding two weeks and may be subject to recall bias. Water samples were collected at a single time point, limiting assessment of temporal variation in contamination. The time elapsed since household flood exposure was not measured; therefore, temporal relationships between flooding, water contamination and diarrhoea could not be assessed. Information on the age and sex of under-five children was not collected and could not be adjusted for in the analysis. Unmeasured confounders, including dietary practices, food hygiene and healthcare-seeking behaviour, may have influenced the findings. Multiple statistical comparisons were performed, increasing the possibility of type I error. As the study was conducted only in flood-displaced communities in Ebonyi State, the findings may not be generalisable to other settings. Despite these limitations, the study provides locally relevant evidence to inform WASH interventions in flood-affected communities.

### Public health implications

These findings emphasise the need for integrated WASH interventions in flood-displaced communities to reduce diarrhoeal risk among under-five children. Priority actions include improving access to safe drinking water, strengthening sanitation, promoting safe child faeces disposal and supporting household water treatment and safe storage [13, 39]. Routine water quality monitoring and integration of WASH measures into flood preparedness and response plans may further reduce exposure and strengthen community resilience.

## Conclusion

This study demonstrates that under-five children in flood-displaced communities in Ebonyi State face considerable diarrhoeal risk in the context of widespread microbial contamination of household drinking water, inadequate sanitation and inconsistent hygiene practices. The findings indicate that post-collection water handling and storage may contribute more to diarrhoeal risk than water source alone. Reducing diarrhoea in these settings will require integrated interventions that strengthen safe water access, sanitation services, hygiene support and environmental management. Priority actions include protecting water sources, promoting household water treatment and safe storage, improving sanitation facilities and supporting safe disposal of children’s faeces. Integrating WASH strategies into flood response planning and routine water quality monitoring may reduce exposure and improve resilience. Future longitudinal studies are needed to clarify causal pathways and seasonal variation.

## Supplementary Information

Supplementary Material 1.

Supplementary Material 2.

Supplementary Material 3.

Supplementary Material 4.

Supplementary Material 5.

## Data Availability

The datasets generated and analysed during the current study are available from the corresponding author on reasonable request.

## Abbreviations

AOR: Adjusted Odds Ratio
CFU: Colony-forming units
CI: Confidence interval
E. coli: Escherichia coli
LGA: Local Government Area
SPSS: Statistical Package for the Social Sciences
WASH: Water, sanitation and hygiene
WHO: World Health Organization
