# Supplementary material for: Flood-related WASH disruption, point-of-use drinking water contamination and diarrhoea among under-five children in flood-affected communities in Ebonyi State, Nigeria
Source: BMC Public Health. 2026 Aug 15;26:3318. doi: 10.1186/s12889-026-29000-w (PMC13753408; doi:10.1186/s12889-026-29000-w)
Supplement: Supplementary file 1 [file 12889_2026_29000_MOESM1_ESM.docx]

**Additional File 1 — Study Questionnaire**

**Flood-related WASH disruption, point-of-use drinking water contamination and diarrhoea among under-five children in flood-affected communities in Ebonyi State, Nigeria**

**CONSENT SECTION**

Good day. My name is ____________________. I am part of a research team conducting a study on flood-related WASH disruption, point-of-use drinking water contamination and diarrhoea among under-five children in flood-affected communities in Ebonyi State, Nigeria. The purpose of this study is to understand how flood-related disruption affects household WASH conditions, point-of-use drinking water contamination and diarrhoea among children under five years in these communities. Your household has been selected because it lives in this community and has at least one child under five years of age. Your participation is voluntary. You may choose not to answer any question or stop the interview at any time without any consequence. The information you provide will be kept confidential and used only for research purposes.

**Do you agree to participate in this study?**
a. No ☐ *(Thank respondent and stop interview)* b. Yes ☐ *(Continue)*

**SECTION A: SOCIODEMOGRAPHIC CHARACTERISTICS**

**A1. Community**
a. Enohia Itim ☐ b. Kpoghirikpo ☐ c. Azuoto-Okpuitumo ☐ d. Onakpoke ☐ e. Inyimagu ☐ f. Igbudu ☐

**A2. Sex of respondent**
a. Male ☐ b. Female ☐

**A3. Age of respondent (years)**
a. 18–29 ☐ b. 30–39 ☐ c. 40–49 ☐ d. 50–59 ☐ e. 60 and above ☐

**A4. Relationship to under-five child**
a. Mother ☐ b. Father ☐ c. Grandparent ☐ d. Other caregiver ☐ e. Household head (non-parent) ☐

**A5. Highest education level completed**
a. No formal education ☐ b. Primary ☐ c. Secondary ☐ d. Tertiary ☐ e. Other ☐

**A6. Main occupation**
a. Farming ☐ b. Trading ☐ c. Civil service ☐ d. Artisan ☐ e. Unemployed ☐ f. Other ☐

**A7. Total number of household members**
a. 1–2 ☐ b. 3–4 ☐ c. 5–6 ☐ d. 7 and above ☐

**A8. Total number of children under five years in the household**
a. 1 ☐ b. 2 ☐ c. 3 ☐ d. 4 and above ☐

**A9. Type of current dwelling**
a. Temporary shelter ☐ b. Mud/thatched house ☐ c. Block/brick house ☐ d. Mixed structure ☐ e. Other ☐

**A10. Main roofing material**
a. Thatch/grass ☐ b. Zinc/iron sheet ☐ c. Concrete/tile ☐ d. Other ☐

**SECTION B: FLOOD EXPOSURE AND FLOOD-RELATED DISRUPTION**

**B1. Was this household directly affected by the most recent flood event?**
a. No ☐ b. Yes ☐

**B2. What was the severity of the impact of the most recent flood on this household?**
a. Mild ☐ b. Moderate ☐ c. Severe ☐

**B3. Was your main drinking water source affected by flooding?**
a. No ☐ b. Yes, partially ☐ c. Yes, severely ☐

**B4. Was your household toilet/latrine affected by flooding?**
a. No ☐ b. Yes, partially ☐ c. Yes, severely ☐ d. No toilet before flood ☐

**B5. Was household access to drinking water disrupted because of flooding?**
a. No ☐ b. Yes ☐

**B6. Did flooding cause visible stagnant water around the dwelling or compound?**
a. No ☐ b. Yes ☐

**B7. Did this household have to leave its usual dwelling because of the most recent flood event?**
a. No ☐ b. Yes ☐

**B8. Where did the household stay during the displacement period?**
a. Camp/shelter ☐ b. School building ☐ c. Relative/friend's house ☐ d. Temporary structure/open field ☐ e. Other ☐ f. Not applicable ☐

**B9. What was the duration of displacement?**
a. 1–5 days ☐ b. 6–10 days ☐ c. 11–20 days ☐ d. 21 days and above ☐

**SECTION C: HOUSEHOLD WATER ACCESS, TREATMENT, STORAGE AND HANDLING**

**C1. What is the main source of drinking water currently used by this household?**
a. Piped water into dwelling/yard ☐ b. Borehole/tube well ☐ c. Protected well ☐ d. Unprotected well ☐ e. Rainwater ☐ f. River/stream ☐ g. Tanker/vendor ☐ h. Sachet/bottled water ☐ i. Other ☐

**C2. Is the main drinking water source within the household compound?**
a. No ☐ b. Yes ☐

**C3. How long does it usually take to go, collect water and return?**
a. Less than 15 minutes ☐ b. 15–30 minutes ☐ c. 31–60 minutes ☐ d. More than 60 minutes ☐

**C4. Is the main drinking water source currently functional?**
a. Always functional ☐ b. Sometimes functional ☐ c. Rarely functional ☐

**C5. Does this household treat drinking water before drinking?**
a. No ☐ b. Yes ☐

**C6. What is the main method used to treat drinking water?**
a. None ☐ b. Boiling ☐ c. Chlorination ☐ d. Filtration ☐ e. Sedimentation ☐ f. Cloth straining ☐ g. Other ☐

**C7. What type of container is mainly used to store drinking water?**
a. Covered bucket/container with lid ☐ b. Open bucket/container ☐ c. Jerry can ☐ d. Clay pot ☐ e. Other ☐

**C8. Is the main drinking water storage container covered at the time of visit?**
a. No ☐ b. Yes ☐

**C9. How is water usually taken from the storage container?**
a. Tap/spout ☐ b. Pouring ☐ c. Dipping with utensil/cup ☐ d. Dipping by hand ☐ e. Other ☐

**C10. How often is the drinking water storage container cleaned?**
a. Daily ☐ b. Weekly ☐ c. Occasionally ☐ d. Never ☐

**SECTION D: SANITATION AND CHILD FAECES DISPOSAL**

**D1. What type of toilet facility does this household mainly use?**
a. Flush/pour flush toilet ☐ b. Ventilated improved pit latrine ☐ c. Pit latrine with slab ☐ d. Pit latrine without slab ☐ e. Hanging toilet ☐ f. Bucket toilet ☐ g. No facility/open defecation ☐ h. Other ☐

**D2. Is the toilet facility shared with other households?**
a. No ☐ b. Yes ☐

**D3. Is the toilet facility currently functional and usable?**
a. No ☐ b. Yes ☐

**D4. If the toilet is not usable, where do household members mostly defecate?**
a. Bush/open field ☐ b. Water body/drain ☐ c. Neighbour's toilet ☐ d. Temporary/shared toilet ☐ e. Not applicable ☐ f. Other ☐

**D5. How are faeces of children under five usually disposed of?**
a. Put/rinsed into toilet/latrine ☐ b. Buried ☐ c. Thrown into refuse dump ☐ d. Left on open ground ☐ e. Thrown into drain/water body ☐ f. Child uses toilet/latrine directly ☐ g. Other ☐

**SECTION E: HOUSEHOLD HYGIENE CONDITIONS**

**E1. Is there a designated place for handwashing in the household?**
a. No ☐ b. Yes ☐

**E2. Was water available at the handwashing place at the time of visit?**
a. No ☐ b. Yes ☐

**E3. Was soap or detergent available at the handwashing place at the time of visit?**
a. No ☐ b. Yes ☐

**E4. Do household members usually wash hands after defecation?**
a. No ☐ b. Yes ☐

**E5. Do household members usually wash hands after cleaning child faeces?**
a. No ☐ b. Yes ☐

**E6. Do household members usually wash hands before preparing food?**
a. No ☐ b. Yes ☐

**E7. Do household members usually wash hands before feeding a child?**
a. No ☐ b. Yes ☐

**E8. Do household members usually wash hands before eating?**
a. No ☐ b. Yes ☐

**E9. Do household members usually wash hands after handling waste?**
a. No ☐ b. Yes ☐

**SECTION F: UNDER-FIVE CHILD AND DIARRHOEA MODULE**

*Enumerator instruction: Complete this section for one selected index child. If more than one under-five child is present in the household, select the youngest child.*

**F1. Age of selected child in completed months**
a. 0–11 months ☐ b. 12–23 months ☐ c. 24–35 months ☐ d. 36–47 months ☐ e. 48–59 months ☐

**F2. Sex of selected child**
a. Male ☐ b. Female ☐

**F3. Has the selected child had diarrhoea in the last 2 weeks?**
a. No ☐ b. Yes ☐

**F4. If yes, for how many days did the diarrhoea last?**
a. 1 day ☐ b. 2 days ☐ c. 3 days ☐ d. 4 days and above ☐

**F5. Was treatment sought for the diarrhoea episode?**
a. No ☐ b. Yes ☐ c. Not applicable ☐

**F6. Where was treatment sought?**
a. Health facility ☐ b. Pharmacy/chemist ☐ c. Traditional healer ☐ d. Home treatment only ☐ e. Other ☐ f. Not applicable ☐

**F7. Was ORS given during the diarrhoea episode?**
a. No ☐ b. Yes ☐ c. Not applicable ☐

**SECTION G: OBSERVATION CHECKLIST**

*Enumerator instruction: Complete this section through direct observation only. Do not ask the respondent. Tick only what you observe.*

**G1. Main drinking water storage container observed**
a. Uncovered ☐ b. Covered ☐

**G2. Main drinking water storage container appears visibly clean**
a. No ☐ b. Yes ☐

**G3. Handwashing place observed**
a. No ☐ b. Yes ☐

**G4. Soap observed at handwashing place**
a. No ☐ b. Yes ☐

**G5. Sanitation facility observed**
a. Improved and functional ☐ b. Unimproved but functional ☐ c. Damaged/non-functional ☐ d. No facility observed ☐

**G6. Visible signs of environmental contamination near dwelling (faeces, stagnant water, refuse)**
a. No ☐ b. Yes ☐

**SECTION H: POINT-OF-USE DRINKING WATER SAMPLE RECORD**

*Enumerator instruction: Complete this section after collecting and labelling the household drinking water sample. Items H2–H5 are completed in the laboratory.*

**H1. Was a household drinking water sample collected?**
a. No ☐ b. Yes ☐

**H2.** ***Escherichia coli*** **contamination status**
a. Negative (0 CFU/100 mL) ☐ b. Positive (≥1 CFU/100 mL) ☐

**H3. Total coliform contamination status**
a. Negative (0 CFU/100 mL) ☐ b. Positive (≥1 CFU/100 mL) ☐

**H4.** ***Escherichia coli*** **risk category**
a. 0 CFU/100 mL (Low risk) ☐ b. 1–10 CFU/100 mL (Medium risk) ☐ c. 11–100 CFU/100 mL (High risk) ☐ d. >100 CFU/100 mL (Very high risk) ☐

**H5. Total coliform risk category**
a. 0 CFU/100 mL (Low risk) ☐ b. 1–10 CFU/100 mL (Medium risk) ☐ c. 11–100 CFU/100 mL (High risk) ☐ d. >100 CFU/100 mL (Very high risk) ☐

**Definition:** Diarrhoea is defined as the passage of three or more loose or watery stools within a 24‑hour period during the two weeks preceding the survey.s

**END OF INTERVIEW**

Interview start time: ________________ Interview end time: ________________

Completed by interviewer: ________________ Checked by supervisor: ________________
